# The impact of β-adrenergic signaling on radioresistance and anti-tumor immunity

**DOI:** 10.1186/2051-1426-3-S2-P267

**Published:** 2015-11-04

**Authors:** Mark Bucsek, Lauren Evans, Timothy Winslow, Bonnie L Hylander, Anurag Singh, Elizabeth Repasky

**Affiliations:** 1 Roswell Park Cancer Institute, Buffalo, NY, USA

## 

Our laboratory has demonstrated that mildly cool housing temperatures, (a stress known to be mediated by sympathetic nerve activity and β-adrenergic receptors) can significantly enhance the incidence, growth, and metastasis of tumors in murine models by suppressing anti-tumor immunity [1]. Furthermore, we have shown that mild cool stress also decreases the sensitivity of cancer cells to several types of chemo- and targeted therapeutics [2]. These data strongly suggest that baseline responses of mouse models of tumor growth and tumor immunology may be significantly influenced by housing temperature and the degree of adrenergic stress experienced by tumor bearing mice. We have now become interested in dissecting the mechanisms by which increased adrenergic stress signaling resulting from cool housing conditions impacts anti-tumor immunity and the response of tumors to therapeutics. To investigate the effects of increased adrenergic signaling on anti-tumor immunity, we used β-AR antagonists (β-blockers) to block β-AR signaling resulting from mild cold stress induced by housing temperatures. The addition of a β-blocker to these mice significantly delayed tumor growth, and recapitulated the increased tumor growth control observed in mice housed at thermoneutrality where cold stress is alleviated. However, β-AR blockade did not alter tumor growth in mice housed at thermoneutrality compared to vehicle controls, suggesting that β-AR signaling is responsible for the impairment of anti-tumor immunity in chronically cold stressed mice. To further investigate the effects of β-AR signaling on suppressing anti-tumor immunity, we repeated these experiments in immunodeficient SCID mice and demonstrated that β-blockers had no effect on tumor growth in mice regardless of housing temperature. We also examined the effects of adrenergic stress signaling on radio-resistance. We addressed this question by stimulating β-ARs on Pan02 (murine) and Mia-PACA2 (human) pancreatic tumor cells and performed clonogenic assays. Those cells treated with a β-agonist exhibited improved survival and proliferation at varying doses of radiation when compared to untreated controls similar to our earlier studies using chemotherapy. In summary, β-adrenergic receptor activity appears to play a fundamental role in the regulation of both anti-tumor immunity as well as intrinsic therapeutic sensitivity. Therefore, strategic combinations of β-receptor antagonists with chemo-radiation therapy, or immunotherapy may provide significantly improved tumor control. This work was supported by the Peter T. Rowley Breast Cancer Research Grant and the Harry J. Lloyd Charitable Trust.

